# Depression in primary TKA and higher medical comorbidities in revision TKA are associated with suboptimal subjective improvement in knee function

**DOI:** 10.1186/1471-2474-15-127

**Published:** 2014-04-11

**Authors:** Jasvinder A Singh, David G Lewallen

**Affiliations:** 1 Medicine Service and Center for Surgical Medical Acute care Research and Transitions (C-SMART), Birmingham VA Medical Center, Birmingham, AL, USA; 2 Department of Medicine at School of Medicine, and Division of Epidemiology at School of Public Health, University of Alabama, Birmingham, AL, USA; 3 Department of Orthopedic Surgery, Mayo Clinic College of Medicine, Rochester, MN, USA; 4 University of Alabama at Birmingham, Faculty Office Tower 805B, 510 20th, Street S, Birmingham, AL 35294, USA

**Keywords:** Total knee arthroplasty, Knee function, Functional limitation, Primary, Arthroplasty, Joint replacement, Outcomes, Patient-reported outcomes

## Abstract

**Background:**

To characterize whether medical comorbidities, depression and anxiety predict patient-reported functional improvement after total knee arthroplasty (TKA).

**Methods:**

We analyzed the prospectively collected data from the Mayo Clinic Total Joint Registry for patients who underwent primary or revision TKA between 1993–2005. Using multivariable-adjusted logistic regression analyses, we examined whether medical comorbidities, depression and anxiety were associated with patient-reported subjective improvement in knee function 2- or 5-years after primary or revision TKA. Odds ratios (OR), along with 95% confidence intervals (CI) and p-value are presented.

**Results:**

We studied 7,139 primary TKAs at 2- and 4,234 at 5-years; and, 1,533 revision TKAs at 2-years and 881 at 5-years. In multivariable-adjusted analyses, we found that depression was associated with significantly lower odds of 0.5 (95% confidence interval [CI]: 0.3 to 0.9; p = 0.02) of ‘much better’ knee functional status (relative to same or worse status) 2 years after primary TKA. Higher Deyo-Charlson index was significantly associated with lower odds of 0.5 (95% CI: 0.2 to 1.0; p = 0.05) of ‘much better’ knee functional status after revision TKA for every 5-point increase in score.

**Conclusions:**

Depression in primary TKA and higher medical comorbidity in revision TKA cohorts were associated with suboptimal improvement in index knee function. It remains to be seen whether strategies focused at optimization of medical comorbidities and depression pre- and peri-operatively may help to improve TKA outcomes. Study limitations include non-response bias and the use of diagnostic codes, which may be associated with under-diagnosis of conditions.

## Background

Total knee arthroplasty (TKA) is a very successful surgery for patients with end-stage arthritis. TKA is associated with significant improvements in pain, function and quality of life [1]. Related to the obesity epidemic, increasing longevity and the expansion of TKA indications to both younger and older patients, the annual incidence of TKA is increasing at an exponential rate [2,3]. Post-TKA functional limitations constitute a significant problem. We need a better understanding of factors associated with failure to improve index knee function after TKA.

Previous studies examining the effect of medical and psychological comorbidity on knee function after TKA have provided contradictory results. Some studies found that higher medical comorbidity was associated with poorer function [4-6], but others reported no association [7-11]. Similarly, some studies reported that depression was associated with poor functional outcomes after TKA [12-14], but others did not [15,16]. While the reasons for these contradictory findings are unclear, there is clearly a lack of consistent evidence of association of medical and psychological comorbidity with knee function after TKA. Not surprisingly, a recent systematic review of psychological factors affecting outcomes of knee or hip arthroplasty concluded “…strong evidence was found that preoperative depression had no influence on postoperative functioning” [14]. Four key limitation of most previous studies were that: (1) they consisted of small sample sizes and were likely underpowered; (2) multivariable-adjustment for potential confounders was not done in all studies, thereby increasing the possibility of bias; (3) they provided a mean change in function score at the cohort level (averaging of excellent, good and poor results), which is difficult to extrapolate to patient level benefit, varying in their improvement in function after TKA; and (4) very few studies included patients with revision TKA. An easier way to understand arthroplasty results is to examine the proportion of patients who achieve a clinically meaningful improvement in function, reported only rarely in arthroplasty studies [17]. Such information can be very helpful to patients and policy makers. Thus, we need better-designed studies. The aim of this study was to examine whether medical and psychological comorbidity at the time of TKA associated with a clinically meaningful function improvement after TKA. We hypothesized that higher medical comorbidity, depression and anxiety at the time of TKA, will each be independently associated with poorer patient-reported subjective functional outcome after primary and revision TKA.

## Methods

We describe the methods and results of this observational study as recommended in the Strengthening of Reporting in Observational studies in Epidemiology (STROBE) statement [18].

### Data source and study population

We used prospectively collected data from the Mayo Clinic Total Joint Registry. The Mayo Clinic Institutional Review Board approved the study and waived the need for patient consent. The Mayo Clinic Total Joint Registry captures data for all patients undergoing TKA using a validated standardized questionnaire, the Mayo knee questionnaire, that has construct validity and reproducibility [19]. Mayo knee questionnaire includes questions assessing pain and function, similar to the Knee Society Scale [20], the most commonly used outcome instrument in TKA patients. The Mayo knee questionnaire was administered to patients during an in-person clinic visit, by mail or by a phone call by trained, dedicated registry staff, at 2- and 5-year time-points post-arthroplasty. Patients were included in this study if they had undergone a primary or revision TKA during 1993–2005 and completed the patient questionnaire at 2- or 5-years post-TKA [21].

### Outcome of interest

Improvement in self-reported subjective knee function compared to the preoperative status was the outcome of interest. It was assessed with a single question: Compared to your condition before your knee surgery, how would you rate your knee function? There were four possible responses: much better, better, same, worse. Patient responses were categorized into ‘much better’ and ‘better’ categories versus the reference category comprising of ‘same’, or ‘worse’. This was based on the assumption that most patients aim and expect to achieve ‘much better’ knee function after TKA, although some may be satisfied with ‘better’ knee function. Thus, both constitute clinically meaningful improvements. Same or worse knee function after TKA, an elective surgical procedure, is clearly undesirable. This question has been used previously to assess patient outcomes in knee and hip arthroplasty populations [21].

### Predictors of interest and covariates

The main predictors of interest assessed were medical and psychological comorbidity at the time of index TKA. Medical comorbidity was measured using Deyo-Charlson index, a validated measure of comorbidity [22]. Deyo-Charlson index consists of a weighted scale of 17 comorbidities (including myocardial infarction, congestive heart failure, peripheral vascular disease, cerebrovascular disease, dementia, chronic pulmonary disease, connective tissue disease, peptic ulcer disease, mild liver disease, diabetes, diabetes with end-organ damage, hemiplegia, moderate or severe renal disease, tumor without metastasis, leukemia, lymphoma, moderate or severe liver disease, metastatic solid tumor and AIDS), expressed as a summative score. It is the most commonly used comorbidity measure in the medical literature and is associated with important outcomes such as mortality, hospitalization and outpatient utilization in populations similar to our cohort [22-24]. The presence of anxiety or depression was assessed based on the presence of the respective ICD-9-CM codes at the time of index TKA, as in previous studies [25-28].

We adjusted the analyses for important covariates and confounders, previously shown to be associated with TKA outcomes (demographics, diagnosis etc.) [10,29-33] or hypothesized to impact outcome, such as distance from medical center due to higher complexity of referred patients versus local patients and differences in expectations which is associated with TKA outcomes [34-36]. Data on these covariates/confounders were obtained from the joint registry and institutional clinical databases. Multivariable-adjusted analyses included the following covariates and confounders:

(1) Demographics: age, categorized as ≤60, >60-70, >70-80, >80, as previously [29,37]; gender; and body mass index (BMI), categorized as normal or underweight, <25, overweight, 25–29.9, obese, 30–34.9, very obese, 35–39.9, or extremely obese, ≥40 as previously, as per WHO classification [38];

(2) American Society of Anesthesiology (ASA) Physical Status score, categorized as class I-II vs. III-IV, as previously [26,39], a validated measure of peri-operative mortality and immediate post-operative morbidity [40];

(3) Distance from medical center (0–100 miles, >100-500 miles, >500 miles), categorized as previously [26,30], calculated by using zip codes and country codes from the patients’ registration records at the time of surgery (if available) or at present;

(4) Operative diagnosis: osteoarthritis, rheumatoid/inflammatory arthritis, or other (avascular necrosis, fracture etc.) for primary TKA; loosening/wear/osteolysis, dislocation/bone or prosthesis fracture/instability/non-union or failed prior arthroplasty with components removed/infection for revision TKA; and

(5) Implant fixation: cemented or uncemented/hybrid, for primary TKA only.

### Statistical analyses

We used univariate logistic regression models to assess the crude (unadjusted) association between medical comorbidity, anxiety and depression and the improvement in knee function 2- and 5-years after primary or revision TKA. Multivariable-adjusted models were used to decrease confounding bias by including all pre-specified covariates significantly associated with outcome and potential confounders. Multivariable-adjusted models included Deyo-Charlson index, anxiety, depression, age, gender, BMI, ASA class, operative diagnosis, distance from the medical center (for both primary and revision TKA) and implant fixation (primary TKA only). Odds ratios (OR), 95% confidence intervals (CI), and p-values were reported. A p-value ≤0.05 was considered statistically significant. All regression analyses used a generalized estimating equations (GEE) approach to adjust the standard errors for the correlation between observations on the same subject due to both knees having been replaced and/or multiple operations on the same knee. We used Statistical Package for the Social Sciences (SPSS) version 19.0 (Chicago, IL) to perform analyses.

### IRB approval

This study was approved by the Mayo Clinic Institutional Review Board and all investigations were conducted in conformity with ethical principles of research.

## Results

We studied 7,139 primary TKAs at 2- and 4,234 at 5-years and 1,533 revision TKAs at 2- and 881 at 5-years. For the primary TKA 2-year cohort, the mean age was 68 years, 18% were 60 years or younger, 9% had BMI of 40 kg/m^2^ or more and 56% were women. Eleven percent had depression, 6% had anxiety, 8% had heart disease, mean Deyo-Charlson index was 1.2, 98% implants were uncemented and osteoarthritis was the underlying diagnosis in 94% (Table 1). Other demographic and clinical characteristics are provided in Table 1. Survey response rates for primary or revision TKA were 65% and 57% at 2-years and 57% and 48% at 5 years, respectively. For primary TKA, men, older age and a diagnosis of osteoarthritis had higher likelihood and patients with higher ASA class, higher Deyo-Charlson score and living >500 miles from the medical center had a lower likelihood of responding to the survey (Table 2). Similar characteristics were associated with non-response in the revision TKA group (Table 2).

**Table 1:** Clinical and demographic characteristics of study cohorts

	**Primary TKA**		**Revision TKA**	
	**2-year (n = 7,139)**	**5-year (n = 4,234)**	**2-year (n = 1,533)**	**5-year (n = 881)**
**Mean age (±standard deviation)**	68 ± 10	68 ± 10	69 ± 10	69 ± 10
**Men/Women (%)**	44%/56%	45%/55%	49%/51%	51%/49%
**Age groups (%)**				
**≤60 yrs**	18%	18%	20%	20%
**>60-70 yrs**	35%	37%	29%	31%
**>70-80 yrs**	38%	38%	42%	41%
**>80 yrs**	8%	7%	9%	8%
**Body mass index (%)**				
**<25 kg/m**^**2**^	13%	13%	13%	14%
**25-29.9 kg/m**^**2**^	35%	36%	36%	39%
**30-34.9 kg/m**^**2**^	29%	43%	29%	27%
**35-39.9 kg/m**^**2**^	14%	7%	14%	14%
**≥40 kg/m**^**2**^	9%	7%	7%	5%
**American Society of Anesthesiologists (ASA) cass**				
**Class I-II**	58%	58%	50%	53%
**Class III-IV**	42%	41%	50%	47%
**Deyo-Charlson index, mean (±standard deviation)**	1.2 ± 1.9	1.1 ± 1.9	1.0 ± 1.7	1.0 ± 1.4
**Deyo-Charlson comorbidities (%)**				
**Myocardial infarction**	5%	5%	4%	4%
**Congestive heart failure**	4%	3%	4%	2%
**Peripheral vascular disease**	5%	4%	3%	2%
**Cerebrovascular disease**	7%	7%	7%	6%
**Dementia**	0.2%	0.1%	0.2%	0.1%
**Chronic Obstructive Pulmonary Disease (COPD)**	11%	10%	9%	9%
**Ulcer disease**	8%	9%	7%	7%
**Mild liver disease**	2%	2%	1%	2%
**Diabetes**	9%	8%	10%	7%
**Diabetes with organ damage**	2%	2%	2%	2%
**Hemiplegia**	0.3%	0.2%	0.4%	0.3%
**Moderate/severe renal disease**	6%	4%	4%	2%
**Moderate/severe liver disease**	0.4%	0.2%	0.5%	0.5%
**Metastatic solid tumor**	3%	3%	1%	1%
**AIDS**	0%	0%	0.1%	0.1%
**Rheumatologic disease**	7%	8%	8%	8%
**Other cancer**	14%	13%	10%	7%
**Psychological comorbidity (%)**				
**Anxiety**	6%	5%	5%	3%
**Depression**	11%	8%	8%	5%
**Distance from medical center**				
**<100 miles**	53%	51%	32%	34%
**100-500 miles**	39%	41%	57%	54%
**>500 miles**	8%	8%	11%	12%
**Implant fixation (cement status)**				
**Uncemented**	98%	99.7%	0%	0%
**Cemented/hybrid**	2%	0.3%	100%	100%
**Operative diagnosis**				
**Osteoarthritis**	94%	93%		
**Rheumatoid arthritis**	4%	4%		
**Loosening/wear/osteolysis**			57%	61%
**Dislocation/fracture/instability/non-union**			22%	20%
**Failed prior arthroplasty with components removed/infection**			21%	19%
**Other**	2%	3%		

**Table 2:** Non-responder characteristics

	**2-yr primary TKA**		**5-yr primary TKA**		**2-yr revision TKA**		**5-yr revision TKA**	
	**Events for non-responders (3818/ 10957)**	**OR (95%)) CI)**	**Events for non-responders (3170/7404)**	**OR (95%)) CI)**	**Events for non-responders (1162/2695)**	**OR (95%)) CI)**	**Events for non-responders (961/1842)**	**OR (95%)) CI)**
**Gender**								
Women	2184/6161 (35.4%)		1860/4191 (44.4%)		621/1402 (44.3%)		509/944 (53.9%)	
Men	1634/4796 (34.1%)	0.94 (0.86,1.03)	1310/3213 (40.8%)	**0.86**^**‡**^** (0.78,0.96)**	541/1293 (41.8%)	1.10 (0.94, 1.29)	452/898 (50.3%)	0.87 (0.72,1.05)
**Age groups n (%)**								
≤60 yrs	841/2154 (39%)		728/1473 (49.4%)		283/587 (48.2%)		241/413 (58.4%)	
>60-70 yrs	1273/3804 (33.5%)	**0.79**^**‡ **^**(0.69,0.89)**	1065/2641 (40.3%)	**0.69**^**‡ **^**(0.60,0.80)**	365/816 (44.7%)	0.87 (0.70,1.08)	281/555 (50.6%)	0.73* (0.56,0.96)
>70-80 yrs	1387/4121 (33.7%)	**0.79**^**‡ **^**(0.70,0.89)**	1142/2759 (41.4%)	**0.72**^**‡ **^**(0.63,0.83)**	391/1034 (37.8%)	0.65^‡^ (0.53,0.81)	350/714 (49%)	**0.69**^**‡ **^**(0.53,0.89)**
>80 yrs	317/878 (36.1%)	0.88 (0.74,1.05)	235/531 (44.3%)	0.81 (0.65,1.01)	123/258 (47.7%)	0.98 (0.72,1.32)	89/160 (55.6%)	0.89 (0.61,1.31)
**BMI Categorized**								
≤24.9	514/1474 (34.9%)		452/1018 (44.4%)		172/375 (45.9%)		136/258 (52.7%)	
25-29.9	1287/3766 (34.2%)	0.97 (0.84,1.11)	1061/2586 (41%)	0.87 (0.74,1.02)	375/925 (40.5%)	0.80 (0.63,1.03)	310/655 (47.3%)	0.81 (0.60,1.09)
30-39.9	1644/4712 (34.9%)	1.00 (0.87,1.15)	1346/3169 (42.5%)	0.92 (0.79,1.08)	485/1149 (42.2%)	0.86 (0.68,1.09)	423/784 (54%)	1.05 (0.79,1.41)
≥40	355/960 (37%)	1.10 (0.91,1.32)	299/602 (49.7%)	1.24 (0.99,1.55)	123/229 (53.7%)	1.37 (0.98,1.91)	89/136 (65.4%)	1.70* (1.09,2.66)
**ASA**								
1-2	2021/6136 (32.9%)		1771/4238 (41.8%)		505/1270 (39.8%)		432/899 (48.1%)	
3-4	1772/4778 (37.1%)	**1.20**^**‡ **^**(1.10,1.31)**	1388/3129 (44.4%)	1.11* (1.00,1.23)	651/1414 (46%)	**1.29**^**‡ **^**(1.10,1.51)**	523/933 (56.1%)	**1.38**^**‡ **^**(1.15,1.66)**
**Deyo-Charlson index (5 point increase)**		**1.30**^**‡ **^**(1.17,1.44)**		1.07 (0.93,1.22)		1.16 (0.93,1.44)		**1.63**^**‡ **^**(1.21,2.20)**
**Income**								
>$45 K	1035/3099 (33.4%)		720/1665 (43.2%)		236/548 (43.1%)		163/304 (53.6%)	
≤$35 K	699/2098 (33.3%)	1.00 (0.87,1.14)	736/1841 (40%)	0.87 (0.75,1.02)	254/595 (42.7%)	1.98 (0.77,1.25)	243/488 (49.8%)	0.86 (0.64, 1.15)
>$35 K-$45 K	1347/4044 (33.3%)	1.00 (0.89,1.11)	1058/2541 (41.6%)	0.94 (0.82,1.07)	450/1050 (42.9%)	0.99 (0.80,1.23)	363/677 (53.6%)	1.00 (0.76,1.32)
**Distance**								
0-100 miles	1785/5454 (32.7%)		1443/3523 (41%)		390/865 (45.1%)		284/571 (49.7%)	
>100-500 miles	1435/4166 (34.4%)	1.08 (0.98,1.19)	1218/2871 (42.4%)	1.06 (0.95,1.19)	602/1449 (41.5%)	0.87 (0.72,1.03)	535/993 (53.9%)	1.18 (0.95, 1.46)
>500 miles or Non-US	476/1017 (46.8%)	**1.81**^**‡ **^**(1.55,2.11)**	382/709 (53.9%)	**1.68**^**‡ **^**(1.40,2.03)**	129/293 (44%)	0.96 (0.73,1.27)	103/200 (51.5%)	1.07 (0.77, 1.50)
**Underlying diagnoses**								
Inflammatory arthritis	172/428 (40.2%)		155/344 (45.1%)					
Osteoarthritis	3480/10190(34.2%)	0.77* (0.62,0.97)	2872/6794 (42.3%)	0.89 (0.70,1.14)				
Other	166/338 (49.1%)	1.44* (1.05,1.96)	143/266 (53.8%)	1.42* (1.00,2.01)				
Loosening/wear or osteolysis					584/1453 (40.2%)		475/1015 (46.8%)	
Dislocation, bone or prosthesis fracture, instability, non-union					254/597 (42.5%)	1.10 (0.91,1.34)	216/393 (55%)	**1.39**^**‡ **^**(1.10,1.76)**
Failed prior arthroplasty with components removed or infection					324/645 (50.2%)	1.50^‡^ (1.24,1.82)	270/434 (62.2%)	**1.87**^**‡ **^**(1.48,2.36)**

^*^P < 0.05; ^‡^p < 0.01, ^†^p < 0.001.

All other p-values are ≥0.05, unless indicated as above.

### Depression, anxiety and Improvement in Knee Function after Primary TKA

At 2-years after primary TKA, 87% reported much better and 10% better knee function compared to preoperatively, and at 5-years, 85% and 10%, respectively. At 2-year follow-up, depression was associated with lower odds of much better knee function (p < 0.01) in univariate analyses (Table 3) as well as lower odds of 0.5 of much better knee function (p = 0.02) after multivariable-adjustment (Table 4). Anxiety was not associated with subjective knee function improvement at 2-years (p ≥ 0.50). At 5-years, depression or anxiety were not significantly associated with knee function improvement (Table 4).

**Table 3:** **Univariate association of comorbidity with overall knee status**^**a **^**at 2- and 5-years after Primary TKA**

		**2-year**			**5-year**		
**Variable**	**Overall knee status**^**a**^	**Overall knee status n/N (%)**	**Odds ratio (95% CI)**	**p-value**	**Overall knee status n/N (%)**	**Odds ratio (95% CI)**	**p-value**
Deyo-Charlson^b^	Better	Not applicable	1.0 (0.7, 1.6)	0.88	Not applicable	1.1 (0.7, 1.6)	0.72
Deyo-Charlson^b^	Much better	Not applicable	0.7 (0.5, 1.1)	0.10	Not applicable	0.8 (0.6, 1.1)	0.14
Anxiety: No	Better	299/3,115 = 9.6%	1.0		481/5076 = 9.5%	1.0	
Yes	Better	24/163 = 14.7%	1.1 (0.5, 2.2)	0.87	40/351 = 11.4%	1.0 (0.5, 1.8)	0.94
Anxiety: No	Much better	2657/3,115 = 85.3%	1.0		4407/5076 = 86.8%	1.0	
Yes	Much better	127/163 = 77.9%	0.6 (0.3, 1.2)	0.14	295/351 = 84.0%	0.8 (0.5, 1.3)	0.37
Depression: No	Better	290/3,008 = 9.6%	1.0		450/4851 = 9.3%	1.0	
Yes	Better	33/270 = 12.2%	0.7 (0.4, 1.2)	0.15	71/576 = 12.3%	1.1 (0.7, 1.8)	0.62
Depression: No	Much better	2572/3,008 = 85.5%	1.0		4222/4851 = 87.0%	1.0	
Yes	Much better	212/270 = 78.5%	**0.5 (0.3, 0.8)**	**<0.01**	480/576 = 83.3%	0.8 (0.5, 1.3)	0.35

^a^Reference category for overall knee status was a patient response of same or worse index knee status; ^b^per 5-point increase.

**Table 4:** **Multivariable-adjusted**^**a **^**odds of Overall Knee status at 2- and 5-years following Primary TKA or Revision TKA**

	**Overall knee status at 2 years**		**p-value**	**Overall knee status at 5 years**	**p-value**
	**Odds ratio (95% CI)**			**Odds ratio (95% CI)**	
**Primary TKA**					
Deyo-Charlson index (5-point increase)					
	Better	1.0 (0.6 to 1.7)	0.90	1.0 (0.6 to 1.5)	0.94
	Much better	0.9 (0.6 to 1.3)	0.47	0.8 (0.6 to 1.2)	0.25
Anxiety					
	Better	1.3 (0.6 to 2.8)	0.58	0.9 (0.5 to 1.8)	0.83
	Much better	0.9 (0.4 to 1.8)	0.71	0.8 (0.5 to 1.5)	0.50
Depression					
	Better	0.6 (0.3 to 1.2)	0.17	1.1 (0.7 to 2.0)	0.61
	Much better	**0.5 (0.3 to 0.9)**	**0.02**	0.9 (0.5 to 1.4)	0.55
**Revision TKA**					
Deyo-Charlson index (5-point increase)					
	Better	1.5 (0.8 to 2.7)	0.21	0.4 (0.2 to 1.1)	0.07
	Much better	1.2 (0.7 to 2.1)	0.48	**0.5 (0.2 to 1.0)**	**0.05**
Anxiety					
	Better	1.3 (0.5 to 3.3)	0.60	1.8 (0.4 to 8.0)	0.45
	Much better	0.9 (0.4 to 2.0)	0.75	0.6 (0.1 to 2.9)	0.56
Depression					
	Better	1.0 (0.5 to 2.0)	0.98	1.1 (0.4 to 3.2)	0.86
	Much better	0.6 (0.4 to 1.2)	0.17	0.5 (0.2 to 1.4)	0.21

^a^Multivariable model additionally adjusted for age to gender to BMI to American Society of Anesthesiologist (ASA) class to distance from medical center to operative diagnosis to implant fixation (cement status) to Deyo-Charlson index to anxiety and depression.

### Depression, anxiety and improvement in knee function after revision TKA

At 2-years after revision TKA, 65% reported much better knee function and 20% better knee function compared to preoperatively, and at 5-years, 63% and 21%, respectively. In univariate analyses at 5-years after revision TKA, depression was associated with lower odds of much better knee function (p = 0.05) (Table 5). In multivariable-adjusted analyses, this was no longer significant (p = 0.17; Table 4). At 5-years, in multivariable-adjusted analyses, neither depression nor anxiety were associated with knee function.

**Table 5:** Univariate association of comorbidity with overall knee status at 2- and 5-years after Revision TKA

		**2-year**			**5-year**		
**Variable**	**Overall knee status category**^**a**^	**Overall knee status n/N (%)**	**Odds ratio (95% CI)**	**p-value**	**Overall knee status n/N (%)**	**Odds ratio (95%) CI)**	**p-value**
Deyo-Charlson index^b^	Better	Not applicable	1.4 (0.8, 2.4)	0.24	Not applicable	0.5 (0.2, 1.1)	0.08
Deyo-Charlson index^b^	Much better	Not applicable	1.2 (0.7, 1.9)	0.50	Not applicable	0.6 (0.3, 1.2)	0.13
Anxiety: No	Better	241/1,257 = 19.2%	1.0		151/727 = 20.8%	1.0	
Yes	Better	18/58 = 31.0%	1.6 (0.7, 3.6)	0.28	8/18 = 44.4%	2.0 (0.5, 7.6)	0.33
Anxiety: No	Much better	827/1,257 = 65.8%	1.0		465/727 = 64.0%	1.0	
Yes	Much better	31/58 = 53.4%	0.8 (0.4, 1.7)	0.54	7/18 = 38.9%	0.6 (0.1, 2.2)	0.40
Depression: No	Better	231/1,212 = 19.1%		1.0	147/707 = 20.8%	1.0	
Yes	Better	28/103 = 27.2%	1.1 (0.6, 2.1)	0.67	12/38 = 31.6%	1.0 (0.4, 2.3)	0.91
Depression: No	Much better	802/1,212 = 66.2%			455/707 = 64.4%	1.0	
Yes	Much better	56/103 = 54.4%	0.7 (0.4, 1.1)	0.13	17/38 = 44.7%	**0.4 (0.2, 1.0)**	**0.05**

^a^Reference category for overall knee status was a patient response of same or worse index knee status; ^b^per 5-point increase.

### Medical comorbidity and improvement in knee function after primary TKA

In univariate analyses, at 2-year follow-up, Deyo-Charlson index was associated with a statistically non-significant trend with much better knee function (p = 0.10) (Table 3). This was no longer significant after multivariable-adjustment, (p = 0.47) (Table 4). At 5-years, Deyo-Charlson index was not significantly associated with knee function improvement (p = 0.25; Table 2).

### Medical comorbidity and improvement in knee function after revision TKA

In univariate analyses at 2 and 5-years after revision TKA, Deyo-Charlson had a non-statistically significant association with much better knee function (p = 0.50 and 0.13; Table 5). In multivariable-adjusted analyses, higher Deyo-Charlson index was associated with significantly lower odds of 0.5 of much better knee function (p = 0.05; Table 4) and a trend towards significantly lower odds of 0.4 of better knee function (p = 0.07; Table 4) at 5-years. No significant associations were noted at 2-years (p > 0.20).

## Discussion

In this study we found that psychological and medical comorbidity were associated with less optimal improvement in knee function after TKA. Specifically, depression was associated with suboptimal knee function improvement at 2-years after primary TKA and higher medical comorbidity score with suboptimal knee function improvement at 5-years after revision TKA.

An interesting finding from our study was the association of depression with suboptimal improvement in index knee function 2-years after primary TKA. Several factors may contribute. Depressed patients are less likely to successfully complete rehabilitation therapy [41,42] that is required post-TKA. They may not follow-up with their surgeon regularly due to concomitant depression and may have worse post-operative pain, which may impact adherence with rehabilitation therapy. Optimal physical rehabilitation after TKA is the key to best results after TKA [43,44]. The absence of this association in primary TKA at 5-years may be either due to a smaller sample size making it underpowered analysis or due to “catching up” by patients with depression after 2-years. The differences in findings between primary and revision TKA may be due to differences in patient characteristics (depression, mean Deyo-Charlson index), the underlying diagnosis and in the rate of complications between primary and revision TKA.

Two recent studies reported that depression was associated with poor functional outcomes after primary TKA [12,13], while other studies failed to confirm this finding [15,16]. Two studies examined function only up to 1-year [12,15], one study at 2-years [16] and one at 5-years [13]. Most studies had small sample size, making them underpowered and at risk of missing a significant association. By analyzing a large sample and performing multivariable-adjusted analyses, our study adds to this body of knowledge related to association of depression with improvement in knee function after TKA. Three key differences between our study and the previous studies are that we used a large sample and our outcome was joint-specific and can be interpreted as a patient-level clinically meaningful outcome. This is in contrast to the use of mean scores on lower limb-specific instrument (Western Ontario McMaster osteoarthritis Index, WOMAC) or lower-limb specific/knee-specific hybrid outcome (such as Knee society score, KSS) in previous studies. Our study extends and confirms previous findings from the positive studies of depression and poor functional outcome. Our finding of no association of anxiety with functional improvement outcomes is important and confirms a previous similar finding in a study with 5-year follow-up [13]. This may be related to smaller sample size at 5-years.

We found that higher medical comorbidity (on Deyo-Charlson index) was significantly associated with suboptimal improvement in knee function 5-years after revision TKA. Previous studies have shown that diabetes and hypertension are associated with higher post-arthroplasty complication rates [45]. Poorer functional outcome associated with higher comorbidity may be partially due to higher post-operative complication rates. A higher comorbidity may also interfere with optimal adherence to physical rehabilitation. In those with primary TKA, evidence is contradictory with some studies finding an association of higher medical comorbidity with poorer function [4-6] and others no such association [7-11]. We did not note any significant association of higher comorbidity at baseline with 2-year outcomes. It is possible that a longer follow-up allows for a more significant impact of comorbidity on TKA outcomes compared to a shorter follow-up, since chronic diseases get worse with longer disease duration, in general. In absence of any previous studies examining patient-level meaningful improvements, these findings are novel and need confirmation in future studies. Studies of improvement of knee function after TKA are important. A $12 million research grant 2010 by the Agency for Healthcare Research and Quality (AHRQ) to study factors associated with functional outcomes and complications after joint replacement is strongly supportive [46]. Our study adds to this growing area of research by studying comorbidity factors as risk factors for poor patient-reported knee function improvement.

Our study has several limitations. Non-response may have biased our findings. Survey responders had characteristics previously shown to be associated with better outcomes (male gender, older age, a diagnosis of osteoarthritis, lower ASA class, lower Deyo-Charlson score, shorter distance to medical center), but it is unclear how the non-response bias might influence the association of depression and medical comorbidity with function outcomes. A lower response rate at 5-years compared to 2-years makes these findings more prone to bias. Since both anxiety and depression were captured based on presence of a diagnostic code, and psychological comorbidities may be under-recognized and under-diagnosed, it is likely that we missed some cases. This might have biased our estimates towards null, and we may have missed some important associations of anxiety and depression with outcomes. A retrospective study design did not allow us to have confirmation of depression/anxiety diagnosis by examination by a psychologist of psychiatrist. However, the prevalence of depression is similar to the 9-15% reported in studies using validated instruments for depression [47-49]. Whether the “much better” is truly different from “better” response on this ordinal scale can not be determined in this study; however, this ordinal response is similar to other validated ordinal scales, commonly used in health outcome assessments [50,51]. Recall bias should be considered while interpreting these results; patients may have over- or under-estimated the functional improvements, and therefore the direction of impact of this bias on our study findings is unclear. Several study strengths must also be noted. We included a large sample size with adequate number of events to study the question of interest, used validated measures (questionnaire, Deyo-Charlson index), performed multivariable-adjusted analyses, examined both 2- and 5-year outcomes in primary and revision TKA and provided results for a clinically meaningful joint-specific functional improvement outcome.

## Conclusion

In summary, in this study using a U.S. institutional joint registry, we found that preoperative depression was associated with less improvement in index knee function 2-years after primary TKA and higher preoperative Deyo-Charlson index was associated with suboptimal improvement in knee function 5-years after revision TKA. These findings clarify the role of medical and psychological comorbidity in functional improvement in the index knee after TKA. Patients with depression may benefit by optimization of behavioral and medical therapy for depression prior to and after primary TKA. Similarly, closer management of medical comorbidities may have an impact on functional outcomes after revision TKA. Patients with higher medical comorbidity load and/or depression should be warned about suboptimal knee function outcome. Future results from the ongoing AHRQ-funded U.S. registry study should help to identify additional factors associated with functional outcomes after TKA [46].

## Abbreviations

TKA: Total knee arthroplasty; BMI: Body mass index; ASA: American Society of Anesthesiology Physical Status score; OR: Odds ratio; CI: Confidence interval.

## Competing interests

There are no financial conflicts related directly to this study. JAS has received research and travel grants from Takeda and Savient; and consultant fees from Savient, Takeda, Regeneron and Allergan. DGL has received royalties/speaker fees from Zimmer, Orthosonic and Osteotech, has been a paid consultant and owns stock in Pipeline Biomedical and his institution has received research funds from DePuy, Stryker, Biomet and Zimmer.

## Authors’ contributions

JAS: Study conception and design, development of study protocol methods and analyses, review of statistical analyses, drafting, critical revisions and submission of the manuscript, approval of the final manuscript version. DGL: Review and revision of study design and study protocol, statistical analyses, critical revisions and approval of the final manuscript version. Both authors read and approved the final manuscript.

## Pre-publication history

The pre-publication history for this paper can be accessed here:

http://www.biomedcentral.com/1471-2474/15/127/prepub
